# 

**Published:** 1891-12

**Authors:** 


### CONTENTS.

ORIGINAL COMMUNICATIONS—

Thirty-two Unselected Abdominal Sections with Tables. By Thomas Opie, M. D., Baltimore, Md ..... 577

Ophthalmia Neonatorum. By Frank Trester Smith, A. M., M. D., Chattanooga, Tenn ..... 602

Synopsis of Remarks on Systemic Infection from Gonorrhœa. By J. Bedford Brown, M. D., Alexandria, Va ..... 607

Blennorrhœa. By James T. Jelks, M. D., Hot Springs, Ark ..... 612

SOCIETY REPORTS—

Tri-State Medical Society of Alabama, Georgia and Tennessee ..... 615

CORRESPONDENCE—

Letter from J. H. Ridley, M. D., Huntsville, Ala ..... 629

Letter from S. T. Overstreet, M. D., Live Oak, Fla ..... 630

EDITORIAL—

Medical Education ..... 631

The History of Syphilis ..... 635

SELECTIONS ..... 637-639

BOOK REVIEWS ..... 640

INDEX TO ADVERTISEMENTS ..... Page xxxiii

